# Subtotal Ovariohysterectomy Following Fetal Maceration and Pyometra in a Maiden Welsh Pony Mare

**DOI:** 10.3390/vetsci9110584

**Published:** 2022-10-23

**Authors:** Rory Nevard, Raphael Labens, Cyril P Stephen

**Affiliations:** 1 School of Agricultural, Environmental and Veterinary Sciences, Charles Sturt University, Wagga Wagga, NSW 2678, Australia; 2 Gulbali Institute for Agriculture, Water and the Environment, Charles Sturt University, Wagga Wagga, NSW 2678, Australia

**Keywords:** equine, maiden, maceration, pyometra, ovariohysterectomy, complications

## Abstract

**Simple Summary:**

A 16-year-old maiden Welsh pony mare was initially presented for management of suspected fetal mummification, and laminitis. Fetal maceration and pyometra was subsequently diagnosed due to death and retention of a mid-term fetus. Transcervical removal of fetal remnants was unsuccessful despite repeated attempts. Surgical intervention via ovariohysterectomy was attempted and resulted in previously unreported complications. This case reports aims to highlight different techniques and expected complications when managing these rare cases in mares.

**Abstract:**

Fetal maceration and pyometra were diagnosed in a 16-year-old maiden Welsh pony mare. Due to anatomical and surgical difficulties encountered throughout treatment, previously reported techniques including both per-vaginum removal of fetal remnants and ovariohysterectomy were attempted and resulted in novel complications. Fetal bones were unable to be removed completely transcervically due to failure of cervical dilation despite repeated attempts. Subsequent surgical complications such as inadequate exposure of the uterus and incorporation of fetal bones into the uterine stump following ovariohysterectomy also occurred. This case highlights some of the difficulties in managing rare cases of mid-term fetal loss and retention that result in maceration. Ultimately, this may provide useful insights to practitioners when managing similar cases in future.

## 1. Introduction

Fetal maceration is a rare condition in horses that typically occurs after fetal death and retention of a fetus mid-gestation [1]. In contrast to fetal mummification, which usually occurs in a closed sterile environment, maceration is a septic process which involves saprophytic digestion of fetal tissues resulting in putrefaction and softening of the fetus [2]. Often maceration occurs as a result of exposure to bacteria that are responsible for the fetal death or arrive ascendingly through an open cervix [3]. Failure to expel and retention of the fetus after fetal death has been postulated to occur as a result of cervical incompetence, malpositioning of the fetus, and uterine inertia [4], and more recently, neoplasia [5]. Reports of management of fetal maceration in the mare have been described in the literature and these include transcervical removal of fetal bones either manually, via endoscopic assistance, or through serial uterine lavage [2,6,7]. Surgical removal of the uterus (ovariohysterectomy) is a recognized procedure in the horse for unresolved cases of pyometra that are refractory to cervical drainage and antimicrobial therapy [8,9,10,11]. Surgical management of cases that involve fetal maceration specifically in the mare have only been rarely reported in peer-reviewed literature [5,12]. Surgical management of fetal maceration in other species has been reported as a recognized procedure and various techniques have been described [13,14,15]. Surgical complications of ovariohysterectomy in the mare may include peritonitis, hemorrhage, and infection of the uterine stump, which reportedly can be reduced when ensuring complete hysterectomy, reducing abdominal contamination, and securely closing the uterine stump [12]. This case report pertains to a nulliparous mare that underwent an ovariohysterectomy for surgical management of fetal maceration that was refractive to both reproductive and medical procedures.

## 2. Case Description

### 2.1. Presentation

**Figure 1:**
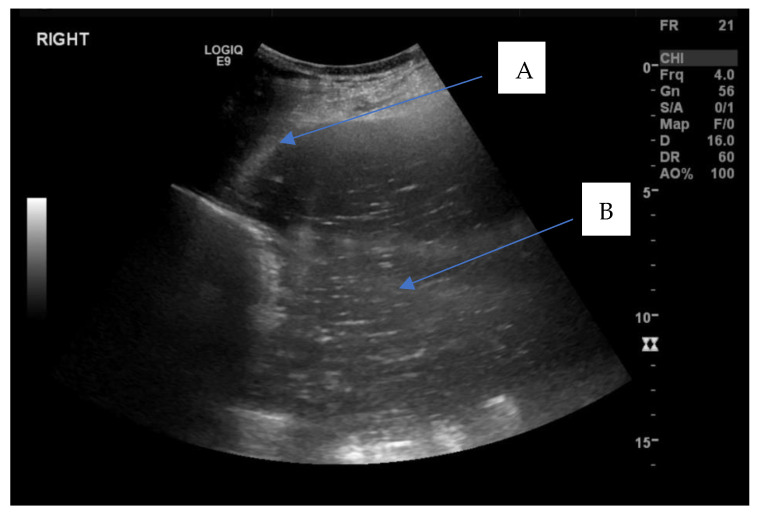
Transabdominal ultrasound showing a distended uterus filled with hyperechoic fluid within uterine lumen. A—uterine wall, B—large volume (>10 cm depth) of mucopurulent intrauterine fluid.

A 16-year-old maiden Welsh pony mare was referred to the Veterinary Clinical Centre, Charles Sturt University, Wagga Wagga for examination of the reproductive tract following an episode of laminitis and suspected fetal mummification during the southern hemisphere spring breeding season. The mare had been paddock bred to a stallion approximately 11 months prior during the previous breeding season in November, and no pregnancy diagnosis had been performed at any stage. The mare’s previous reproductive history was unknown as she was kept in a large paddock for natural mating with a stallion. No previous natural mating or artificial breeding was attempted prior to November the previous year. On presentation, a reproductive examination was performed. The mare had normal perineal conformation, no evidence of vaginal trauma from an attempted foaling and no apparent vulval discharge. Transrectal palpation and ultrasonography of the reproductive tract using a 5 MHz linear transrectal probe (GE LOGIQ^TM^, V2 GE Healthcare, Parramatta, NSW, Austrilia), revealed multiple medium sized follicles (20–25 mm) on both left and right ovaries which both had palpable ovulation fossae. A large distended, fluid-filled uterus was palpated per rectum, and on transrectal ultrasonography, the fluid had medium echogenicity with an increased echogenicity proximal to the cervix. Transabdominal ultrasonography (GE LOGIQ^TM^ S7, GE Healthcare, NSW, Parramatta, Austrilia) confirmed the presence of a distended fluid-filled uterus suggestive of pyometra (Figure 1). 

Vaginoscopic examination using a vaginal speculum (KRUUSE Vaginal Speculum 35 cm, Jørgen Kruuse A/S, Langeskov, Denmark) and palpation per vaginum subsequently revealed a tight cervix that was unable to be manually dilated for evacuation of the contents. 

### 2.2. Treatment

**Figure 2:**
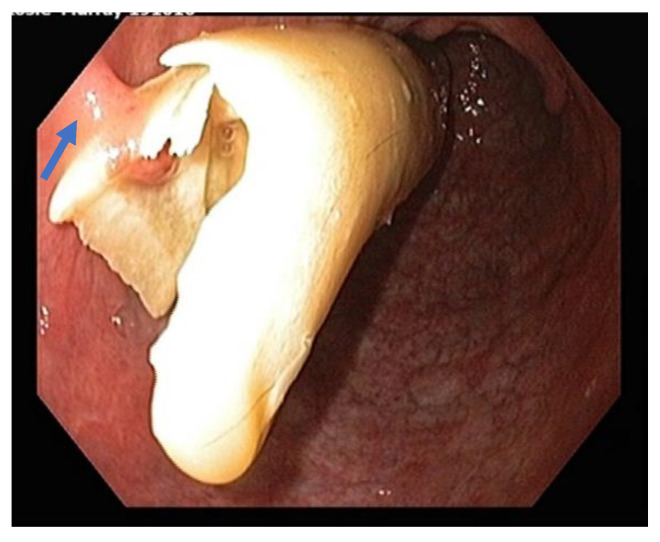
Uteroscopic view of the uterine lumen showing a fetal femur protruding from the endometrium within the right horn of the uterus. A partial adhesion to the endometrium can be seen on the left side of the image (blue arrow).

A blood sample was taken to measure serum progesterone levels and was found to be 2.5 nmol/L (0.78 ng/mL), confirming absence of any luteal tissue. A hematological profile and biochemistry analysis was also performed prior to initial treatment. Major laboratory findings included a mild lymphopenia with mild toxic changes to white cell morphology. Blood biochemistry revealed mildly elevated fibrinogen and serum amyloid A, as well as mild hypertriglyceridemia. The mare was sedated using detomidine hydrochloride (Dozadine, Virbac, 0.01 mg/kg IV, Milperra, NSW, Australia) and butorphanol tartrate (Ilium Butorgesic, 0.01 mg/kg IV, Troy Laboratories Pty Ltd, Glendenning, NSW, Australia) and the perineal region was aseptically prepared with povidone iodine solution. Two hundred micrograms of a crushed misoprostol (PGE1) tablet (Cytotec, Pfizer, New York, NY, USA) mixed with sterile lubricant (KY Jelly, Durex®, Parsippany, NJ, USA ) was applied to the cervix and 10 mg of estradiol benzoate (Bomerol, Bayer Australia, Pymble, NSW, Australia) was administered intramuscularly to facilitate cervical relaxation. Cervical dilation was tested approximately 1 hour after administration of the cervical relaxants, and approximately only 3 cm of dilation was achieved. Uteroscopy was performed using a 1.5 m videoendoscope and the uterus was subsequently insufflated with approximately 300–500 mL of air. Uteroscopy revealed a mucopurulent fluid-filled uterine lumen with fetal skeletal remains embedded within the endometrium at the base of both left and right horns (Figure 2). Removal of the fetal bones was attempted both manually and under endoscopic assistance using an endoscopic snare (15 mm Ratigator Grasping Forcep, AUSTVET Endoscopy, Mount Waverley, VIC, Australia). Both methods were found to be unsuccessful, predominantly due to insufficient cervical dilation (about 3 cm maximum dilation) to facilitate the passage of either the operator’s fingers or the bones themselves. Using a 65 cm 33Fr Foley uterine flush catheter (DLC, Hoppers Crossing, VIC, Australia), and 5 litres of sterile 0.9% sodium chloride solution, uterine lavage was subsequently performed, removing the mucopurulent intrauterine fluid, but fetal bones remained in situ. Facilitation of further cervical relation was attempted 24 hours later, which was unsuccessful. Due to the failed attempts at less invasive transcervical techniques to remove the remnant fetal bones, the decision to undergo surgery was indicated. 

### 2.3. Surgery

**Figure 3:**
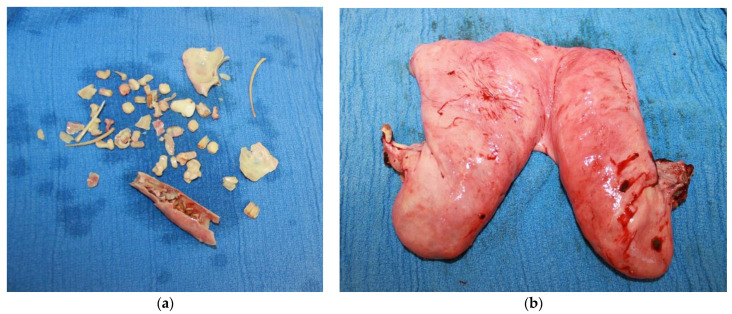
(**a**) Collection of fetal bones which were retrieved from the uterus post ovariohysterectomy, this includes scapulae, rib bones, and vertebral fragments. (**b**) Uterine tissue removed after subtotal ovariohysterectomy.

The mare was initially sedated preoperatively with 12 mg of romifidine (Sedivet®, Boehringer Ingelheim, North Ryde, NSW, Australia) and 30 mg of morphine IV. Prophylactic antimicrobial therapy in the form of penicillin 22 mg/kg IM (Propercillin, Ilium, Glendenning, NSW, Australia), and gentamicin 6.6 mg/kg IV (Gentam 100, Ilium, Glendenning, NSW, Australia) were also administered. The mare was induced with 600 mg of ketamine (Ketamav, Mavlab, Slacks Creek, QLD, Australia) and 15 mg of midazolam (Jurox, Rutherford, NSW, Australia) IV, and subsequently maintained on inhalational anesthesia (desflurane) throughout the duration of surgery. The mare was positioned on the operating table in dorsal recumbency, and the skin from the xiphoid to the mammary gland was clipped and aseptically prepared. A midline laparoscopic approach was undertaken whereby a laparoscope portal was created 2 cm caudal to the umbilicus. Two instrument portals were made on both the right and the left of midline, 10 cm lateral and 3 cm caudal from the laparoscope portal. The abdominal cavity was insufflated with carbon dioxide (CO_2_) to a maximum pressure of 15 mm of Hg. Using the laparoscope (HOPKINS® straight telescope, Karl Storz Endoscopy, Macquarie Park, NSW, Australia), the left ovary was located first and removed using an endoscopic vessel sealing device (Ligasure^TM^ Blunt Tip Laparoscopic Sealer/Divider 44 cm, Valleylab, Boulder, CO, USA). The right ovary was not able to be visualized despite exploration of the abdomen in neutral and Trendelenburg position. Consequently, a ventral midline laparotomy was then performed, with the laparoscopic portal being extended both cranially and caudally to a 25 cm length incision. The right ovary was palpated and a Roeder’s knot was used to ligate the ovarian pedicle with 2 polyglycolic braided sutures (Safil®, B.braun, Bella Vista, NSW, Australia), and the ovary was removed using blind transection. The uterus was further mobilized via transection of the broad ligament using the Ligasure device and exteriorized. The uterine arteries were ligated with USP 2 absorbable multifilament suture (2 Vicryl, Ethicon®, Johnson and Johnson International, North Ryde, NSW, Australia). Three stay sutures were placed, and traction was applied on the remaining uterine tissue to elevate it close to the abdominal incision, and doyen clamps were placed to maintain exposure. Absorbable multifilament suture (2 Vicryl) was then used in a continuous horizontal suture pattern first, and the uterus was then removed distal (i.e., caudal) to the uterine horn bifurcation. The uterine stump was then oversewn using a simple continuous Parker Kerr suture technique. Approximately 8–10 cm of the uterine tissue stump was left in situ. Figure 3 shows the fetal bones and the amount of uterine tissue that was removed during the ovariohysterectomy. The stay sutures were removed, and the uterine stump was examined for any hemorrhage on release. Irrigation and subsequent lavage of the abdomen was performed using 2.7 liters of sterile 0.9% sodium chloride solution. The laparoscopic instrument portals were closed using 2–0 vicryl for the subcutaneous tissue and USP 0 absorbable monofilament suture (Polydioxanone E, Silverglide^TM^, Thornleigh, NSW, Australia) for the skin in a cruciate pattern. The ventral midline laparotomy incision was closed using a three-layer closure consisting of USP 5 vicryl for closure of the linea alba, USP 2 PDS for closure of the subcutaneous tissue using a continuous suture pattern, and USP 0 PDS for skin closure in a Ford interlocking pattern. An abdominal stent was oversewn over the incision, and an adhesive compression bandage (Elastoplast, Zebravet Pty. Ltd., Braeside, VIC, Australia) was applied over the abdomen. 

**Figure 4:**
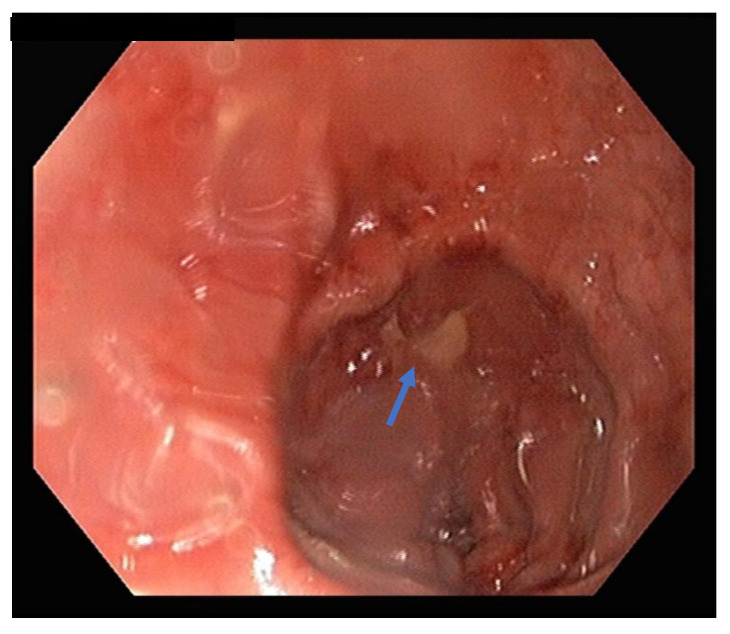
Uteroscopy showing fetal bone (blue arrow) remnant incorporated into the uterine stump closure post-ovariohysterectomy.

Post-surgery, the mare was managed in the hospital. This included intravenous fluid therapy in the form of maintenance isotonic fluids, pain relief (flunixin meglumine 1.1 mg/kg IV q12 hourly), ongoing antimicrobial therapy (procaine penicillin 22 mg/kg IM BID, gentamicin 6.6 mg/kg IV SID) and continued reproductive management including serial sterile uterine lavages using varying volumes of 0.9% sodium chloride solution. Due to the accompanying signs of laminitis, digital cryotherapy using ice boots was commenced and later external coaptation support of the forelimb feet with adjustable raised wedge shoes (NANRIC Ultimate^TM^, Melbourne, VIC, Australia). Throughout treatment the mare remained stable with no indication of severe pain (monitored according to physical examination and clinical picture), inappetence or sepsis. The characteristics of the fluid volumes retrieved via lavage improved well during treatment. Three days post-surgery, the mare underwent a reproductive examination, and it was found that some of the bones remained attached to the uterine stump. Uteroscopy confirmed the presence of four bones now embedded within the endometrium (see Figure 4). Although some of the bones were now retrievable using long grasping forceps, the larger bones still remained in situ due to the incomplete cervical compliance. 

### 2.4. Outcome

The mare was discharged approximately one month after initial presentation and lived for 5 years without any evidence of ongoing acute laminitis or reproductive issues reported by the owner. Following discharge, the mare underwent continual therapeutic podiatry and farriery to mitigate the pedal bone sinking and rotation that occurred during the prior laminitic episode. 

## 3. Discussion

Although the mare indeed survived post-hospitalization, there were a number of unprecedented complications encountered throughout treatment, such as inadequate cervical relaxation, which resulted in failed attempts at transcervical removal of fetal remnants, as well as surgical complications such as inadequate exposure of the uterus and incorporation of fetal remnants into the uterine stump. This account is unique as it is the first reported case in the literature of fetal remnants being incorporated into the caudal stump of the ovariohysterectomy site as a complication in surgical management of fetal maceration and pyometra in the mare. In previous reports, successful resolution of the condition by evacuation of the remnant fetal structures was performed via less invasive transcervical endoscopic techniques and uterine lavage. However, this approach proved to be unsuccessful due to failure of cervical relaxation and incomplete compliance. Previously reported techniques for relaxation of the cervix include pharmacological treatments such as systemic prostaglandin F2α (250 µg cloprostenol) and manual application of prostaglandin E_1_ (crushed 200 µg Cytotec tablet) on the cervix itself [1,16]. Unfortunately, these techniques were not successful in this case. This could possibly have been due to fibrotic changes in the cervix resulting in poor elasticity and failure to dilate, being an older maiden mare. Due to the evidence of a deteriorating clinical picture in the mare, along with failed transcervical evacuation attempts, intervention in the form of surgery was considered. Ovariohysterectomy is a well-recognized procedure in the horse for unresolved cases of pyometra that are refractory to medical management, including cervical drainage and antimicrobial therapy [8,9,10,11]. Despite the reported technical difficulties associated with the procedure, most clinical reports summarize that the prognosis post-surgery can be quite good [12,17]. Surgical management of cases that involve fetal maceration, specifically in the horse, have only been reported in the literature twice [5,12]. However, despite this, surgical management of fetal maceration in other species has been well recognized [13,14,15]. Various approaches to access the uterus have been described including a transvaginal approach via colpotomy [18], standing abdominal approaches including both left and right flank incisions in the cow [12,13,15], and recumbent abdominal celiotomy approaches such as paramedian and ventral midline incisions in mares [8,12] as well as both standing and recumbent laparoscopic approaches [5,11,19,20]. A common issue encountered during this procedure in mares is achieving complete and adequate exposure to the caudal aspect of the reproductive tract [1,12,17]. Similar difficulties were encountered during surgery for this mare, and this was perhaps attributable to both the inherent anatomy and stature of the mare as well as the size and positioning of the uterus itself. Techniques to improve surgical exposure include using stay sutures, right angled clamps, and perhaps even the use of a TA-90 Autosuture instrument (4United States Surgical Corp., Norwalk, CT, USA) as described by Rötting et al. [17]. Other reports describe initial dissection of ovarian structures via intra-abdominal laparoscopic techniques, with subsequent removal/amputation of the uterus caudally via a colpotomy [21] or through the left flank via a paralumbar fossa incision [11]. Removal of the fetus via a hysterotomy without removal of the uterus has also been described in the cow and may be another alternative procedure for consideration in horses [22,23]. Ovariohysterectomy over hysterotomy was chosen in this mare to prevent recurrence of pyometra in future. Unlike in cattle where pyometra is associated with a persistent corpus luteum and closed cervix, the main reason for development of pyometra in mares is due to a problem with cervical drainage because of cervical incompetence. This was an old nulliparous mare, and age-related fibrotic changes likely led to the failure of cervical dilation even after manual and medical interventions. The progesterone level in this mare was baseline (0.78 ng/mL) which ruled out any influence of cervical closure due to increased progesterone levels. Moreover, the influence of progestogens from the feto-placental unit was also ruled out due to absence of fetal membranes. There are other recognized complications associated with partial and/or complete ovariohysterectomies which include peritonitis, infection of the uterine stump, abdominal hemorrhage, and incision site infection [12,17]. Additional considerations as to cost and prognosis may not be completely applicable to every manifestation of these rare cases. In this particular instance, the owners of this mare were not severely financially restricted and a decision to treat this mare was mainly due to their emotional attachment to the animal. 

This case ultimately highlights the difficulties in managing mid-term fetal loss without expulsion and subsequent maceration in the mare. Surgical approaches are indeed indicated in the literature although potential complications need to be considered if this is to be the chosen course of management. Therefore, ongoing hospitalization is a likely sequelae post-surgery and needs to be considered in financial discussions. Other surgical approaches that may improve complete uterine exposure such as those described by Kadic and Bonilla [11], and Gablehouse and Cary [21], may be worth consideration if a similar case were to be encountered by practitioners in the future. 

## 4. Conclusions 

Management of mid-term fetal death and retention can be difficult in the mare and may not always be amenable to medical therapy without surgical intervention. Various techniques have been reported in the literature although there appears to be no consensus in the management of these rare cases. Incomplete cervical relaxation was a major issue encountered during treatment of this maiden pony mare that ultimately led to consideration of surgical management to remove the remaining fetal remnants and compromised uterine tissue. Ensuring complete exposure of the uterus during ovariohysterectomy via a ventral midline laparotomy has limitations and other surgical approaches such as colpotomy may be indicated in future. Despite the encountered complications, such as incorporation of fetal bones in the uterine stump, the mare lived for 5 years post-hospitalization. 

## Data Availability

All data are available on request.
